# Chemical and genetic diversity of *Astragalus mongholicus* grown in different eco-climatic regions

**DOI:** 10.1371/journal.pone.0184791

**Published:** 2017-09-25

**Authors:** Lin Li, Sihao Zheng, Josef A. Brinckmann, Juan Fu, Rui Zeng, Linfang Huang, Shilin Chen

**Affiliations:** 1 Institute of Medicinal Plant Development, Chinese Academy of Medical Sciences & Peking Union Medical College, Beijing, China; 2 Sustainability Department, Traditional Medicinals, Sebastopol, California, United States of America; 3 Southwest University for Nationalities, Chengdu, China; 4 Institute of Chinese Materia Medica, China Academy of Chinese Medical Sciences, Beijing, China; Defence Research and Development Organisation, INDIA

## Abstract

*Astragalus mongholicus* Bunge (Fabaceae) is an important plant source of the herbal drug known as Radix Astragali, which is used worldwide as a medicinal ingredient and a component of food supplement. Russian Federation, Mongolia, Kazakhstan, and China are the main natural distribution areas of *A*. *mongholicus* in the world. However, the quality of medicinal plant varies among different locations. As for *A*. *mongholicus*, limited literature focused on its biodiversity mechanism. Here, we combined the chemometric analysis of chemical components with genetic variation, as well as climatic and edaphic traits, to reveal the biodiversity mechanism of *A*. *mongholicus*. Results showed that the detected chemical, genetic and climatic traits comprehensively contributed to the quality diversity of *A*. *mongholicus*. The eight main chemical components, as well as the inorganic elements of P, B and Na were all significant chemical factors. The precipitation and sunshine duration were the main distinguishing climatic factors. The inorganic elements As, Mn, P, Se and Pb were the distinguishing edaphic factors. The systematic method was firstly established for this medicinal plant in order to illustrate the formation of diversity in terms of quality, and provide scientific evidence for geographic indications and climatic adaptation in production and in the clinical application of herbal medicinal plants.

## Introduction

*Astragalus mongholicus* Bunge (Fabaceae) is the main plant source of the herbal drug Radix Astragali, which is recorded by European Pharmacopoeia (EP, 8.0 version), British Pharmacopoeia (BP, 2013 version), Japanese Pharmacopoeia (JP, 16 version) and Chinese Pharmacopoeia (CP, 2010 version). *A*. *mongholicus* is locally adapted and chemically, climatically and genetically differentiated for traits related to quality. Russian Federation, Mongolia, Kazakhstan and Inner Mongolia, Shanxi and Gansu Provinces in China, are the major natural distribution areas for *A*. *mongholicus* in the world. *A*. *mongholicus* is used not only in the Chinese, Japanese, and Korean systems of medicine but also widely cultivated and used outside of Asia, such as Germany and the United States[1] for its immunostimulant, anti-perspirant, antidiarrheal, anti-diabetic and tonic properties[2]. According to previous studies, the content of astragalosides, calycosin-7-glucoside and formononetin content varied among different habitats, which indicated that different habitats possessed medicinal materials of different composition, quality and strength[3, 4]. This phenomenon was related to biodiversity, local adaptation or ‘geoherbalism’ which involves the use of geo-herbs for higher clinical efficacy[5]. There is a relationship between geographical indications and geoherbalism[6]. In China, geoherbalism can be protected as traditional Chinese medical knowledge under the geographical indication regulatory framework[7]. Generally, geo-herbs have higher quality and are distributed in specific geographic origins with characteristic natural conditions and ecological environments. Furthermore, geo-herbs involve particular techniques for cultivation, harvesting and processing. Therefore, the quality and clinical effects surpass those of the material coming from the same botanical origin but produced in other regions[8]. The content of chemical components and therapeutic effects are the essential differences between the same medicinal materials from geo-producing areas and other locations. The total sugar, reducing sugar and soluble polysaccharide content in Asian ginseng (*Panax ginseng* C.A. Mey., Araliaceae) root samples from fifteen different areas showed variations related to geographic location[9]. The stem of *Dendrobium candidum* Wall. ex Lindl. (Orchidaceae) obtained from six different areas showed significantly different content of mannose and polysaccharide[10]. Several relevant studies have focused on ecological and geographical differences using combined technologies and statistical analyses to determine the biodiversity and geoherbalism mechanism in herbal medicines[11–15].

For *A*. *mongholicus*, studies have tried to explain the differences in the molecular variation and soil properties. However, limited literature accounted for the mechanisms of biodiversity by systematical method and reciprocal correlation based on chemical, genetic, climatic and edaphic traits[16–19]. In the present study, *A*. *mongholicus* was used as a representative medicinal plant to interpret the mechanism of biodiversity and local adaptation that could be attributed to internal (chemical components and genetic variation) and external (climatic and edaphic traits) causes based on chemometric and correlation analysis, which is essential for the geographic indications and further scientific development of herbal medicinal plants.

## Materials and methods

### Materials information

**Fig 1:**
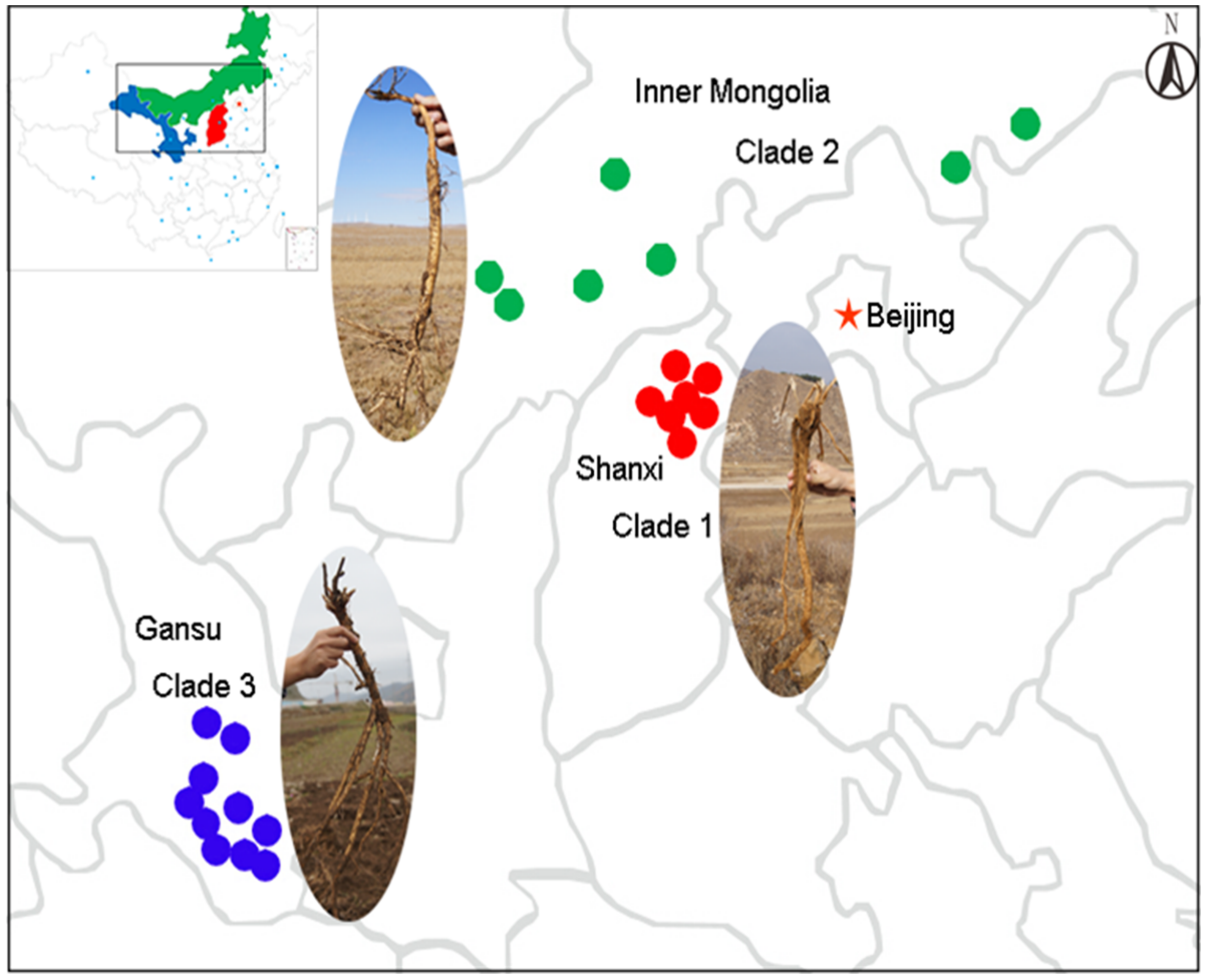
The collection information of *Astragalus mongholicus*. *A*. *mongholicus* were collected for three main producing areas, Inner Mongolia, Shanxi, Gansu province in China. The collection sites and habitat of *A*. *mongholicus* were shown.

The *A*. *mongholicus* samples were collected from three locations, namely, the provinces of Inner Mongolia, Shanxi and Gansu, which are the main producing areas in the People’s Republic of China (S1 Table, Fig 1). The samples were collected on 28 October, 2012 after two-years of cultivation in unified size and quality grade. The raw medicinal materials were cleaned by water, removing the fibrous root and root apex, and then hung to dry in the sun naturally and uniformly. The dried samples were crushed into powder separately for further analysis of components. The samples were handled identically and met the same basic inclusion criteria. All corresponding voucher specimens were deposited to the Herbarium of the Institute of Medicinal Plant Development (IMPLAD) at the Chinese Academy of Medical Sciences in Beijing, China.

### Chemical content determination

The six flavonoid active components (calycosin, ononin, quercetin, calycosin-7-glucoside, kaempferol and formononetin) and astragaloside were quantified by ultra-performance liquid chromatography (UPLC). The chromatographic conditions for determining the six flavonoids were: ACQUITY UPLC BEH C18 column (2.1 mm×100 mm, 1.7 μm; Waters, Milford, MA, USA); column temperature was maintained at 30°C; and the mobile phase consisted of (A) water containing 0.1% formic acid and (B) acetonitrile-isopropyl alcohol (7:3) containing 0.1% formic acid. The elution conditions were optimized as follows: 10%-20% B held for (0–1 min); 20%-25% B held for (1–6 min); 25%-30% B held for (6–8 min); 30%-40% B held for (8–10 min); 40%–100% B held for (10–12 min); 100%-10% B held for (12–13 min); 10% B held for (13–14 min); the injection volume was 2 μL; and the flow rate was set at 0.25 mL/min.

The chromatographic conditions for determining the astragaloside content were: ACQUITY UPLC BEH C18 column (2.1 mm ×100 mm, 1.7 μm; Waters, Milford, MA, USA); the column temperature was maintained at 30°C; the mobile phase was acetonitrile–water (8:2) for isocratic elution; the injection volume was 2 μL; the flow rate was set at 0.25 mL/min; the ELSD condition was 45°C, and the pressure for N_2_ was 25 psi. All experiments were performed in triplicate.

The content of Astragalus polysaccharide was determined by the phenol–sulfuric acid method[20]. The inorganic elements in the herb and soil were determined by inductively coupled plasma atomic emission spectrometry (ICP-AES)[21]. The method mainly referred to the general principles of ICP-AES.

### DNA extraction, PCR amplification and sequencing

The material specimens were dried by natural methods, and 30 mg of dried plant material was used for DNA extraction. Samples were rubbed for 2 min at a frequency of 30 r/s in a FastPrep bead mill (Retsch MM400, Germany). The total genomic DNA was isolated from the crushed material according to the manufacturer’s instructions (Plant Genomic DNA Kit, Tiangen Biotech Co., China). We made the following modifications to the protocol: chloroform was diluted with isoamyl alcohol (24:1), and the buffer solution GP2 was diluted with isopropanol (same volume). The powdered sample, 700 μL of 65°C GP1 and 1 μL of β-mercaptoethanol were mixed for 10–20 s before the mixture was incubated for 60 min at 65°C. Subsequently, 700 μL of a chloroform: isoamyl alcohol mixture was added, and the solution was centrifuged for 5 min at 12, 000 rpm (~13400×*g*). The supernatant was removed and transferred into a new tube before adding 700 μL isopropanol and mixing for 15–20 min. The mixture was centrifuged in CB3 spin columns for 40 s at 12,000 rpm. The filtrate was discarded and 500 μL GD (adding quantitative anhydrous ethanol before use) was added before centrifugation at 12,000 rpm for 40 s. The filtrate was discarded, and 700 μL PW buffer (quantitative anhydrous ethanol was added before use) was used to wash the membrane before centrifugation for 40 s at 12,000 rpm. This step was repeated with 500 μL PW, followed by a final centrifugation step for 2 min at 12,000 rpm to remove the residual wash buffer. The spin column was dried at room temperature for 3–5 min and then centrifuged for 2 min at 12,000 rpm to obtain the total DNA.

General PCR reaction conditions and universal DNA barcode primers were used for the ITS, ITS2, and *psb*A-*trn*H barcodes, as presented in S2 Table. PCR amplification was performed on 25 μL reaction mixtures containing 2 μL of the DNA template (20–100 ng), 8.5 μL of ddH_2_O, 12.5 μL of 2×*Taq* PCR Master Mix (Beijing TransGen Biotech Co., China), and 1/1 μL of the forward/reverse (F/R) primers (2.5 μM). The reaction mixtures were amplified in a 9700 GeneAmp PCR system (Applied Biosystems, USA). Amplicons were visualized by electrophoresis on 1% agarose gels. The purified PCR products were sequenced in both directions by an ABI 3730XL sequencer (Applied Biosystems, USA).

### Climatic data collection

The climatic data were collected from the China meteorological data sharing service system (Website: http://cdc.cma.gov.cn/home.do). The data of 20 climatic factors from 1951 to 2012 were included in our analysis.

### Data analysis

The PCA, one-way ANOVA, and correlation analyses for the chemical, climatic and molecular factors as well as the inorganic elements in the soil were performed with the SPSS for Windows (release version 22.0; SPSS Institute, Cary, NC, USA).

The DNA sequencing peak diagrams were obtained and proofread before the contigs were assembled with CodonCode Aligner 5.0.1 (CodonCode Co., USA). The complete ITS2 sequences were obtained via the HMMer annotation method based on the Hidden Markov model[22]. All of the sequences were aligned using ClustalW. The ML trees were constructed based on the Tamura–Nei model, and bootstrap tests were conducted with 1,000 repeats to assess the confidence of the phylogenetic relationships by MEGA 6.0 software[23].

## Results

### Chemical analysis

**Fig 2:**
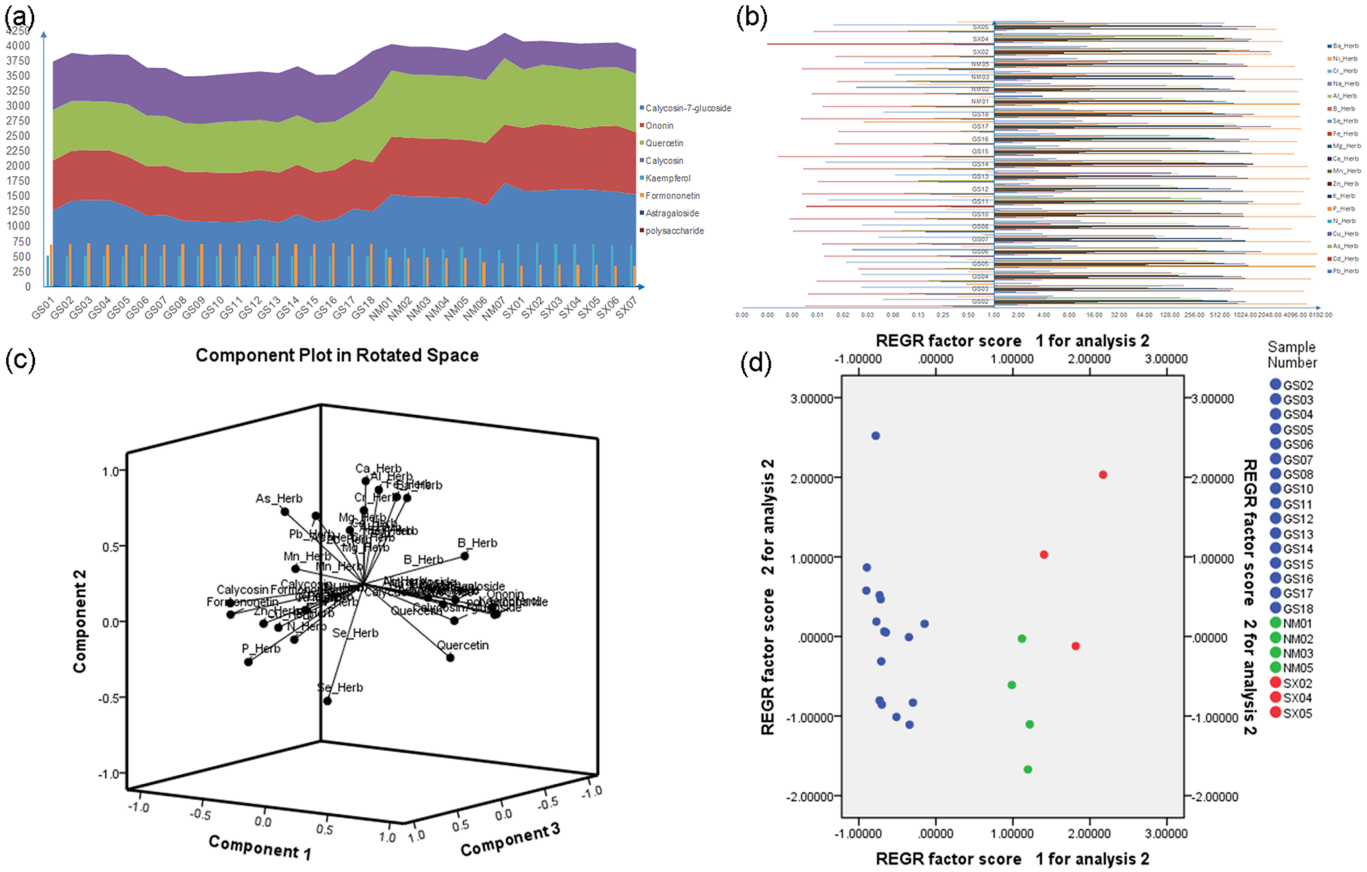
The analysis of chemical components in *Astragalus mongholicus*. The content of main chemical organic constituents and inorganic elements in the root of *A*. *mongholicus* were determined. PCA were performed to analyze the chemical components from different origins. (a) The chemical content of main organic constituents; (b) The chemical content of inorganic elements; (c) The loading diagram of chemical components; (d) The PCA analysis of chemical components from different origins.

The content of the main chemical components in the root of *A*. *mongholicus* obtained from different locations were determined and analyzed (Fig 2). The main chemical organic constituents and inorganic elements were respectively shown in Fig 2(a) and 2(b). The content of eight main constituents was distinct among the different locations, and the samples from Shanxi Province had the highest total content compared with other two origins. For the content of inorganic elements, samples from Gansu Province possessed slightly higher average content than those from the other two locations. Furthermore, the content of toxic metal elements, including Pb, Cd, Cr and As were in the range of the safety values (The Pharmacopoeia of the People’s Republic of China requires that Radix Astragali should contain not-more-than (NMT) 5 ppm of Pb, 0.3 ppm of Cd, 2 ppm of As, 0.2 ppm of Hg, and NMT 20 ppm of Cu). From the loading diagram and PCA analysis, three origins were significantly divided into three parts (Fig 2(c) and 2(d)).

### Genetic variation analysis

**Fig 3:**
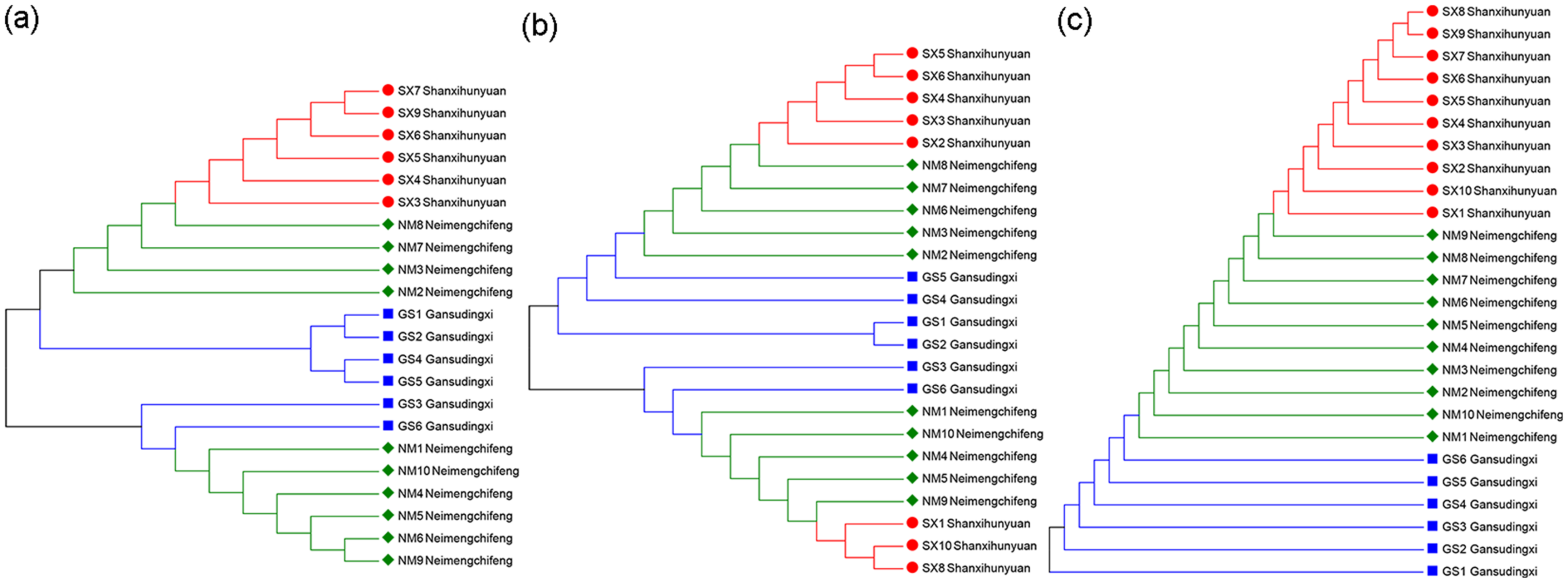
The maximum likelihood (ML) trees of *Astragalus mongholicus* based on DNA barcodes. The ML trees were built by MEGA 6.0 for analyzing the genetic variation of *A*. *mongholicus* from different origins. (a) The ML tree of ITS for *A*. *mongholicus*; (b) The ML tree of ITS2 for *A*. *mongholicus*; (c) The ML tree of psbA-trnH for *A*. *mongholicus*.

Three DNA barcodes of the ITS, ITS2, and *psb*A*-trn*H intergenic region were obtained and used for the analysis of the genetic variation among *A*. *mongholicus* from different origins[24]. Maximum likelihood (ML) trees were constructed based on the Tamura–Nei model (Fig 3). The barcodes could distinguish *A*. *mongholicus* from three different locations. The *psb*A*-trn*H intergenic region performed better than ITS and ITS2 regions. The results showed that *A*. *mongholicus* from different origins possessed genetic variation.

### Climatic and edaphic traits analysis

**Fig 4:**
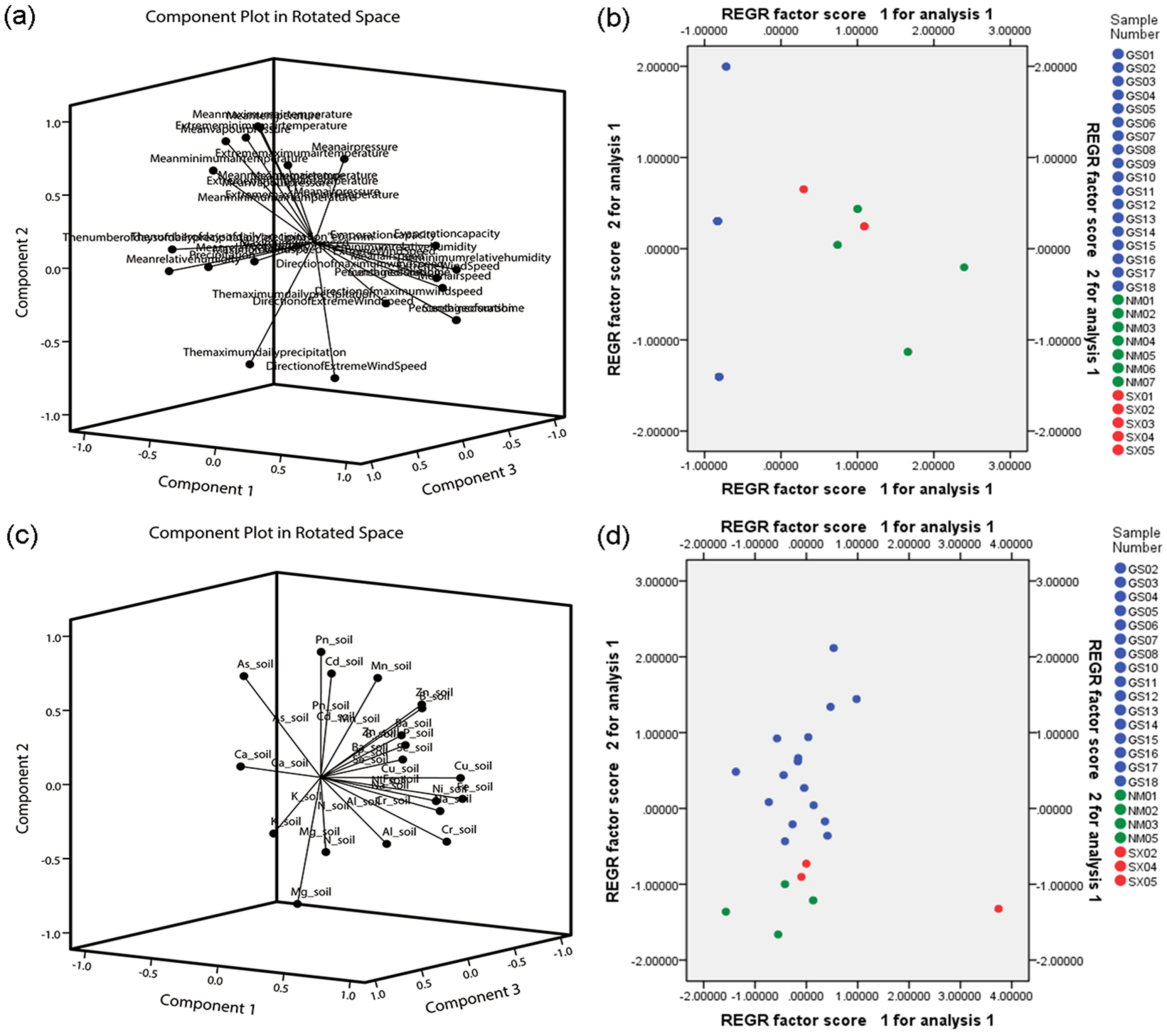
The loading diagram and PCA of climatic and edaphic traits for *Astragalus mongholicus* from different origins. The loading diagram and PCA were conducted by SPSS 22.0. (a) The loading diagram of climatic traits for *A*. *mongholicus*; (b) The PCA of climatic traits for *A*. *mongholicus*; (c) The loading diagram of edaphic traits for *A*. *mongholicus*; (d) The PCA of edaphic traits for *A*. *mongholicus*.

For this study, 20 climatic factors and 19 edaphic factors collected from different locations were analyzed. The loading diagram and PCA were employed (Fig 4), which demonstrated that different origins possessed discriminating climatic and edaphic conditions. The analysis for climatic factors showed that the mean temperature, extreme maximum temperature, maximum wind speed and the maximum daily precipitation distributed around the coordinate axis were the principal component traits that influenced the biodiversity of *A*. *mongholicus* from different origins (Fig 4(a) and 4(b)). The inorganic elements As, Mg, Ca and K were the principal component traits that influenced the biodiversity of different origins in terms of edaphic factors (Fig 4(c) and 4(d)). According to the PCA, Shanxi and Inner Mongolia provinces were more closely clustered than Gansu province, and this result was consistent with the distribution of *A*. *mongholicus* (Fig 1).

### ANOVA analysis

One-way analysis of variance (ANOVA) was performed for the chemical, molecular, climatic and edaphic factors (Table 1). We aimed to analyze each factor and to find the distinguishing factors for the subsequent correlation analysis. As shown in Table 1, compared to ITS region, ITS2 was more significant in molecular factor. The eight main chemical components, as well as the inorganic elements of P, B and Na were all significant chemical factors. The precipitation, mean air speed, sunshine duration, evaporation capacity, extreme wind speed, mean relative humidity, percentage of sunshine, mean minimum air temperature, the number of days of daily precipitation (≥0.1 mm) and the extreme minimum air temperature were the distinguishing climatic factors. The inorganic elements As, Mn, P, Se and Pb were the distinguishing edaphic factors. The results provided a reference for the mutual authentication of the correlation analysis of the four influence factors.

**Table 1:** ANOVA analysis for four factors.

Factors		df	MS	F	*p* value
Chemical factors	Calycosin-7-glucoside	2	471872.708	35.579	**<0.001**
	Ononin	2	162279.693	366.095	**<0.001**
	Quercetin	2	145169.944	64.481	**<0.001**
	Calycosin	2	493694.318	506.155	**<0.001**
	Kaempferol	2	115282.497	1642.381	**<0.001**
	Formononetin	2	379761.624	1016.041	**<0.001**
	Astragaloside	2	0.039	8.265	**0.001**
	polysaccharide	2	103.349	626.505	**<0.001**
	Pb_Herb	2	0.006	0.350	0.709
	Cd_Herb	2	0.000	0.052	0.949
	As_Herb	2	0.006	1.191	0.324
	Cu_Herb	2	1.626	0.305	0.740
	N_Herb	2	0.006	0.743	0.489
	P_Herb	2	11733783.192	11.781	**<0.001**
	K_Herb	2	57037.369	2.041	0.156
	Zn_Herb	2	84.883	0.578	0.570
	Mn_Herb	2	28.627	0.177	0.839
	Ca_Herb	2	519332.317	4.429	**0.026**
	Mg_Herb	2	11664.960	0.844	0.445
	Fe_Herb	2	262706.514	6.138	**0.008**
	Se_Herb	2	0.001	1.328	0.287
	B_Herb	2	317.708	10.834	**0.001**
	Al_Herb	2	60487.214	2.497	0.108
	Na_Herb	2	79135.596	13.586	**<0.001**
	Cr_Herb	2	17.512	3.648	**0.045**
	Ni_Herb	2	1.332	3.023	0.071
	Ba_Herb	2	48.576	6.990	**0.005**
Genetic factors	ITS	2	0.051	0.595	0.563
	ITS2	2	0.000	9.861	**0.002**
Climate factors	Extreme Wind Speed	2	3688.149	25.173	**<0.001**
	Direction of Extreme Wind Speed	2	14513.131	2.153	0.134
	Extreme minimum air temperature	2	22877.700	7.357	**0.003**
	Extreme maximum air temperature	2	1440.476	0.764	0.475
	Precipitation	2	9442427.354	45.174	**<0.001**
	Mean air pressure	2	2606289.956	5.424	**0.010**
	Mean air speed	2	883.835	22.539	**<0.001**
	Mean temperature	2	1568.399	1.035	0.368
	Mean vapour pressure	2	1376.102	3.747	**0.036**
	Mean relative humidity	2	700.708	123.385	**<0.001**
	Mean minimum air temperature	2	12134.097	26.794	**<0.001**
	Mean maximum air temperature	2	1393.867	1.708	0.199
	The number of days of daily precipitation ≥0.1mm	2	789901.491	6.562	**0.004**
	Percentage of sunshine	2	1122.675	58.232	**<0.001**
	Sunshine duration	2	220881643.256	58.956	**<0.001**
	Evaporation capacity	2	200765905.569	31.022	**<0.001**
	Maximum wind speed	2	51.422	0.066	0.936
	Direction of maximum wind speed	2	162.657	0.059	0.943
	The maximum daily precipitation	2	237657.196	4.067	**0.028**
	The minimum relative humidity	2	4519.264	4.931	**0.014**
Edaphic factors	Pn_soil	2	9.665	7.702	**0.003**
	Cd_soil	2	0.000	3.873	**0.038**
	As_soil	2	2.989	16.994	**<0.001**
	Cu_soil	2	12.417	1.508	0.245
	N_soil	2	0.004	1.560	0.235
	P_soil	2	635044.885	9.939	**0.001**
	K_soil	2	2321.627	0.226	0.800
	Zn_soil	2	170.347	4.704	**0.021**
	Mn_soil	2	23076.101	14.662	**<0.001**
	Ca_soil	2	145961142.572	7.570	**0.004**
	Mg_soil	2	1750405.463	4.701	**0.021**
	Fe_soil	2	2142231.607	2.476	0.109
	Se_soil	2	54170670.967	8.971	**0.002**
	B_soil	2	40.524	4.692	**0.021**
	Al_soil	2	3219046.146	1.103	0.351
	Na_soil	2	1463.477	3.044	0.070
	Cr_soil	2	12.166	2.018	0.159
	Ni_soil	2	14.541	3.193	0.063
	Ba_soil	2	613.615	5.149	**0.016**

Significant differences are shown in bold type.

### Correlation analysis and diversity evaluation

**Fig 5:**
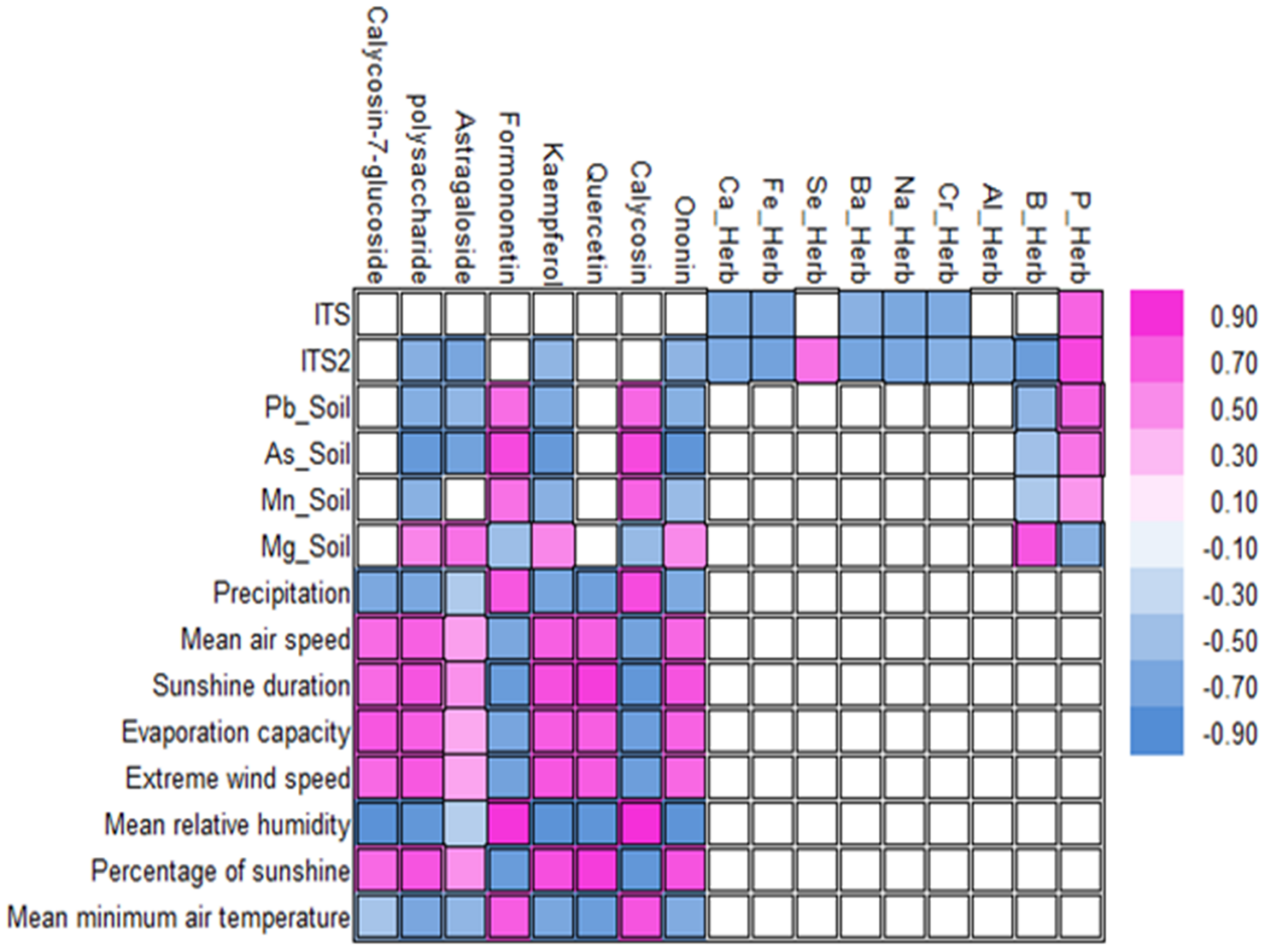
The correlation analysis of chemical components and genetic, climatic and edaphic traits. The correlation analysis was performed based on the loading diagram, PCA and ANOVA analysis of four factors.

Based on the loading diagram, PCA and ANOVA analysis for four factors, the correlation between four factors were conducted (Fig 5). For the chemical and genetic traits, the genetic distance of ITS2 was negatively correlated with the chemical components, except for the inorganic elements of P and Se in the roots, which were positively correlated with ITS2. The genetic distance of ITS was negatively correlated with Ca, Fe, Ba, Na and Cr, but was positively correlated with P. The *psb*A-*trn*H intergenic region showed no significant differences in terms of genetic distance. Thus, we excluded this region in subsequent analysis. For the chemical and climatic factors, the main chemical components calycosin-7-glucoside, polysaccharides, kaempferol, quercetin, and ononin were strongly positively correlated with the climatic traits of mean air speed, sunshine duration, evaporation capacity, extreme wind speed and percentage of sunshine, but these components were strongly negatively correlated with the precipitation, mean relative humidity and mean minimum air temperature. Meanwhile, formononetin and calycosin were strongly negatively correlated with the mean air speed, sunshine duration, evaporation capacity, extreme wind speed and percentage of sunshine, but were strongly positively correlated with the precipitation, mean relative humidity and mean minimum air temperature. Astragaloside was weakly positively correlated with the mean air speed, sunshine duration, evaporation capacity, extreme wind speed and percentage of sunshine but was weakly negatively correlated with the precipitation, mean relative humidity and mean minimum air temperature. For the chemical and edaphic factors, polysaccharide, kaempferol and ononin were strongly positively correlated with the inorganic elements Pb, As and Mn, but were weakly negatively correlated with Mg. Formononetin and calycosin were strongly positively correlated with Pn, As and Mn but weakly negatively correlated with Mg. Calycosin-7-glucoside and quercetin had no correlation with Pb, As, Mn and Mg. The inorganic element of B in the roots was positively correlated with Pb, As and Mn, but negatively correlated with Mg. Conversely, P in the roots was negatively correlated with Mg, but positively correlated with Pb, As and Mn. The inorganic elements in the roots were not correlated with the climatic factors.

## Discussion

Different geographic locations, climatic conditions and edaphic traits may contribute to the different quality levels of medicinal plants. In this study, the *A*. *mongholicus* samples were collected from three main producing areas, namely, Shanxi, Inner Mongolia and Gansu provinces in China. The samples were analyzed in terms of their chemical, genetic, climatic and edaphic traits, which were the main factors of influence corresponding to the quality of herbal material. The eight main active constituents included six flavonoids, astragaloside and Astragalus polysaccharide[25]. The content of the main active constituents showed that *A*. *mongholicus* produced in Shanxi province contained the highest total content. By contrast, the content of the inorganic elements in the roots were not significantly different. Remarkably, the content of Astragaloside was higher in the samples of Inner Mongolia than those from the other two locations. As an isolated active constituent, Astragaloside mainly confers a protective effect against ischemic-heart disease and brain injury. The content of flavonoid and Astragalus polysaccharide were higher in the samples from Shanxi province. Flavonoid and polysaccharide components of *A*. *mongholicus* are mainly used for anti-neoplastic properties and inhibition of atherosclerosis formation. The present results were consistent with those of previous studies[26, 27].

To explore the genetic variation between different locations, we employed three commonly used DNA barcodes in medicinal plants, ITS, ITS2 and *psb*A-*trn*H intergenic region, to analyze *A*. *mongholicus* from three different locations. The ML tree showed that the samples from different locations were divided into different clusters. The results were consistent to a certain degree with those of previous studies[28, 29]. According to the report by Liu *et al*., the ITS sequence for *A*. *mongholicus* from different regions were highly conservative at an intra-specific level. In our study, we confirmed the performance of ITS and its partial sequence of ITS2 in *A*. *mongholicus*. The ML tree of ITS and ITS2 were in substantial agreement, because ITS2 was a part of the ITS sequence. Furthermore, we combined the chloroplast barcodes, *psb*A-*trn*H intergenic region, which performed well compared with ITS and ITS2 sequence in our previous studies[30–32]. The *psb*A-*trn*H intergenic region performed better than the other two barcodes because it divided the samples into three observable clusters. The results indicated that *A*. *mongholicus* from different locations possessed stable genetic variation in the *psb*A-*trn*H intergenic region, which could be used as a molecular marker to distinguish samples from different locations.

In addition, the analysis of climatic and edaphic traits demonstrated that *A*. *mongholicus* from different locations had different climatic and edaphic conditions. The PCA results showed that the three locations were obviously divided into three clusters. Nonetheless, the quality of Radix Astragali is the result of comprehensive influences. The ecological environment, which comprises the climatic and edaphic traits, was the main external cause, whereas the chemical component and genetic variation were the main internal causes that influenced the quality of Radix Astragali. Therefore, we analyzed the correlation between the chemical components and the genetic variation as well as the climatic and edaphic traits.

The ITS2 barcode was strongly negatively correlated with the main chemical components of Astragalus polysaccharide, astragaloside, kaempferol, and ononin. By contrast, ITS was not correlated with the main chemical components. The results indicated that the variation of genetic distance was negatively correlated with the chemical components on a certain level. In terms of the edaphic inorganic element and chemical components, we found that the harmful elements of Pb and As were strongly negatively correlated with the content of Astragalus polysaccharide, astragaloside, kaempferol, and ononin, but were strongly positively correlated with the content of formononetin and calycosin. These results were interesting because several heavy metals, such as Cu and Zn, are essential trace minerals of plants. Meanwhile, some heavy metals, such as Pb, Cd and Hg, are trace minerals that are not necessary for the growth of plants, and a very small amount can cause toxic contamination of the plants[33]. The inorganic element of Mn, which is a transition metal, was strongly negatively correlated with the Astragalus polysaccharide, kaempferol and ononin content, but strongly positively correlated with formononetin and calycosin. The inorganic element Mg was negatively correlated with formononetin and calycosin, but was positively correlated with Astragalus polysaccharide, astragaloside, kaempferol, and ononin. Among the climatic factors and chemical components, the mean air speed, sunshine duration, evaporation capacity, percentage of sunshine and extreme wind speed were strongly positively correlated with the chemical content of calycosin-7-glucoside, polysaccharide, kaempferol, quercetin, ononin, and astragaloside, thereby indicating that the above mentioned climatic traits affected the quality of *A*. *mongholicus*.

## Conclusion

This work proposes a systematic and comprehensive method for estimating the quality of *A*. *mongholicus* from different locations based on internal (chemical component and genetic variation) and external (climatic and edaphic traits) causes. The correlation analysis proved that the genetic, climatic and edaphic traits were similarly closely correlated with the content of the chemical components. This proposed method presents useful data for estimating the quality of *A*. *mongholicus*. Thus, the findings reported here firstly establish a scientific method to clarify the mechanisms of the biodiversity and local adaptation for medicinal plants. Further study should be focused on genome-wide studies, and developing suitable methods for quantitative traits analysis, which is conducive to better understand the biodiversity and local adaptation in plant population, especially for medicinal plants.

## Supporting information

S1 Table Voucher number and location for samples in this study. (DOCX)

S2 Table Primers and PCR reaction conditions for three DNA barcodes. (DOC)
