# Supplementary material for: Chemical and genetic diversity of Astragalus mongholicus grown in different eco-climatic regions
Source: PLoS One. 2017 Sep 25;12(9):e0184791. doi: 10.1371/journal.pone.0184791 (PMC5612462; doi:10.1371/journal.pone.0184791)
Supplement: S1 Table — (DOCX) [file pone.0184791.s001.docx]

S1 Table Voucher number and location for samples in this study.

| Voucher number | Species | Sampling Location |
| --- | --- | --- |
| GS01 | *Astragalus mongholicus* | Gansu Provinces，China |
| GS02 | *Astragalus mongholicus* | Gansu Provinces，China |
| GS03 | *Astragalus mongholicus* | Gansu Provinces，China |
| GS04 | *Astragalus mongholicus* | Gansu Provinces，China |
| GS05 | *Astragalus mongholicus* | Gansu Provinces，China |
| GS06 | *Astragalus mongholicus* | Gansu Provinces，China |
| GS07 | *Astragalus mongholicus* | Gansu Provinces，China |
| GS08 | *Astragalus mongholicus* | Gansu Provinces，China |
| GS09 | *Astragalus mongholicus* | Gansu Provinces，China |
| GS10 | *Astragalus mongholicus* | Gansu Provinces，China |
| GS11 | *Astragalus mongholicus* | Gansu Provinces，China |
| GS12 | *Astragalus mongholicus* | Gansu Provinces，China |
| GS13 | *Astragalus mongholicus* | Gansu Provinces，China |
| GS14 | *Astragalus mongholicus* | Gansu Provinces，China |
| GS15 | *Astragalus mongholicus* | Gansu Provinces，China |
| GS16 | *Astragalus mongholicus* | Gansu Provinces，China |
| GS17 | *Astragalus mongholicus* | Gansu Provinces，China |
| GS18 | *Astragalus mongholicus* | Gansu Provinces，China |
| NM01 | *Astragalus mongholicus* | Inner Mongolia, China |
| NM02 | *Astragalus mongholicus* | Inner Mongolia, China |
| NM03 | *Astragalus mongholicus* | Inner Mongolia, China |
| NM04 | *Astragalus mongholicus* | Inner Mongolia, China |
| NM05 | *Astragalus mongholicus* | Inner Mongolia, China |
| NM06 | *Astragalus mongholicus* | Inner Mongolia, China |
| NM07 | *Astragalus mongholicus* | Inner Mongolia, China |
| NM08 | *Astragalus mongholicus* | Inner Mongolia, China |
| NM09 | *Astragalus mongholicus* | Inner Mongolia, China |
| NM10 | *Astragalus mongholicus* | Inner Mongolia, China |
| SX01 | *Astragalus mongholicus* | Shanxi Provinces，China |
| SX02 | *Astragalus mongholicus* | Shanxi Provinces，China |
| SX03 | *Astragalus mongholicus* | Shanxi Provinces，China |
| SX04 | *Astragalus mongholicus* | Shanxi Provinces，China |
| SX05 | *Astragalus mongholicus* | Shanxi Provinces，China |
| SX06 | *Astragalus mongholicus* | Shanxi Provinces，China |
| SX07 | *Astragalus mongholicus* | Shanxi Provinces，China |
| SX08 | *Astragalus mongholicus* | Shanxi Provinces，China |
| SX09 | *Astragalus mongholicus* | Shanxi Provinces，China |
| SX10 | *Astragalus mongholicus* | Shanxi Provinces，China |
