# Supplementary material for: Chemical and genetic diversity of Astragalus mongholicus grown in different eco-climatic regions
Source: PLoS One. 2017 Sep 25;12(9):e0184791. doi: 10.1371/journal.pone.0184791 (PMC5612462; doi:10.1371/journal.pone.0184791)
Supplement: S2 Table — (DOC) [file pone.0184791.s002.doc]

S2 Table Primers and PCR reaction conditions

| Primer name | Primer sequences（5’-3’） | PCR reaction condition |
| --- | --- | --- |
| ITS2 2F | ATGCGATACTTGGTGTGAAT | 94℃ 5min； |
| 3R | GACGCTTCTCCAGACTACAAT | 94℃ 30s， 56℃ 30s，  72℃ 45s， 40cycles；  72℃ 10min； |
| ITS 4R | TCCTCCGCTTATTGATATGC | 94℃ 5min； |
| 5F | GGAAGTAAAAGTCGTAACAAGG | 94℃ 1min，50℃ 1min，  72℃ 1.5min+3s/cycle，30cycles；  72℃ 7min； |
| *psb*A fwdPA | GTTATGCATGAACGTAATGCTC | 94℃ 4min； |
| *trn*H rev TH | CGCGCATGGTGGATTCACAATCC | 94℃ 30s，55℃ 1min，  72℃ 1min，35 cycles；  72℃ 10min； |
